# Global effects of agriculture on fluvial dissolved organic matter

**DOI:** 10.1038/srep16328

**Published:** 2015-11-06

**Authors:** Daniel Graeber, Iola G. Boëchat, Francisco Encina-Montoya, Carlos Esse, Jörg Gelbrecht, Guillermo Goyenola, Björn Gücker, Marlen Heinz, Brian Kronvang, Mariana Meerhoff, Jorge Nimptsch, Martin T. Pusch, Ricky C. S. Silva, Daniel von Schiller, Elke Zwirnmann

**Affiliations:** 1 Department of Bioscience, Aarhus University, Denmark; 2 Department of Geosciences, Federal University of São João del-Rei, Brazil; 3 Faculty of Natural Resources, Environmental Science Nucleus, Catholic University of Temuco, Chile; 4 Department of Chemical Analytics and Biogeochemistry, Leibniz-Institute of Freshwater Ecology and Inland Fisheries, Germany; 5 Department of Theoretical and Applied Ecology, CURE – Faculty of Sciences, Universidad de la República, Uruguay; 6 Department of Ecosystem Research, Leibniz-Institute of Freshwater Ecology and Inland Fisheries, Germany; 7 Instituto de Ciencias Marinas y Limnologicas, Facultad de Ciencias, Universidad Austral de Chile, Edificio Emilio Pugin, Campus Isla Teja, Valdivia, Chile; 8 Department of Natural Sciences, Federal University of São João del-Rei, Brazil; 9 Department of Plant Biology and Ecology, Faculty of Science and Technology, University of the Basque Country, Spain

## Abstract

Agricultural land covers approximately 40% of Earth’s land surface and affects hydromorphological, biogeochemical and ecological characteristics of fluvial networks. In the northern temperate region, agriculture also strongly affects the amount and molecular composition of dissolved organic matter (DOM), which constitutes the main vector of carbon transport from soils to fluvial networks and to the sea, and is involved in a large variety of biogeochemical processes. Here, we provide first evidence about the wider occurrence of agricultural impacts on the concentration and composition of fluvial DOM across climate zones of the northern and southern hemispheres. Both extensive and intensive farming altered fluvial DOM towards a more microbial and less plant-derived composition. Moreover, intensive farming significantly increased dissolved organic nitrogen (DON) concentrations. The DOM composition change and DON concentration increase differed among climate zones and could be related to the intensity of current and historical nitrogen fertilizer use. As a result of agriculture intensification, increased DON concentrations and a more microbial-like DOM composition likely will enhance the reactivity of catchment DOM emissions, thereby fuelling the biogeochemical processing in fluvial networks, and resulting in higher ecosystem productivity and CO_2_ outgassing.

The environmentally safe operating space of humanity on Earth is limited and some Earth-system processes, such as the nitrogen cycle and the climate system are already beyond their limits^1^. The climate system^2^, as well as the nitrogen and other biogeochemical cycles^3^ are significantly affected by the global carbon transport from soils to freshwaters, and human activities have altered this transport from approximately 1.1 Pg yr^−1^ to 1.9 Pg yr^−1 ^4. This carbon flux is mainly organic^2^, and the largest part of it is DOM^5^. The molecular composition and DON content of this DOM can be heavily altered by changes and intensification in agricultural land use^6^,^7^,^8^.

Changes in quantity and composition of DOM exported from soils can strongly affect the receiving aquatic ecosystems, by changing their metabolism, light regime, as well as by modulating the activity of other chemicals and biological processes^9^,^10^. Furthermore, DOM reactivity in fluvial networks determines the DOM quantity and composition entering marine environments and the carbon reaching the atmosphere^11^,^12^. In fact, a recent study suggests that the outgassing of CO_2_ from fluvial networks and lakes to the atmosphere is approximately twice as high as previously thought and calls for better understanding of the sources of this CO_2_ 13.

Agricultural practices can alter both DOM amount and composition in temperate fluvial networks through alterations of hydrological flow paths, changes of the soil organic matter pool and altered aquatic microbial processing^10^. Relative to pristine, undisturbed catchments, DOM exported from agricultural catchments is often altered in a way which enables it to be potentially more reactive in aquatic ecosystems^6^,^7^,^8^,^14^, and therefore it may increase productivity, outgassing and burial of carbon in fluvial networks^11^,^12^. However, so far it remains unclear if the effect of agriculture is a global phenomenon, relevant to the global carbon cycle and coupled elemental cycles^3^.

To address this issue, we sampled headwater streams draining 45 reference and 75 agricultural catchments situated within five climate zones in the northern and southern hemispheres. The chosen climate zones include some of the largest and most rapidly intensifying areas of agriculture worldwide^15^. We tested, if agricultural land use results in similar effects on fluvial DOM, independent of the global region in which the samples have been taken. Two major types of agriculture, as well as reference catchments were investigated: (i) arable farming with soil tillage, artificial fertilization and with partial drainage (intensive farming), which covers approximately 12% of Earth’s land surface area, (ii) livestock production on permanent grasslands (extensive farming), which covers approximately 26% of Earth’s land surface area^15^ and (iii) pristine, reference catchments with natural vegetation but otherwise similar characteristics as the agricultural catchments. Fluvial DOM samples for intensive farming were taken in headwater catchments situated in northern temperate (lowlands of Germany and Denmark), Mediterranean (North-east Spain), subtropical (grasslands of Uruguay) and tropical (transition zone between the Brazilian Cerrado and the Atlantic Forest) climate. Samples for extensive farming were taken in northern temperate, subtropical and tropical climate, and also in southern temperate climate (lowlands of North Patagonia, Chile). In each of the climate zones, samples were taken in each main season (winter and summer or wet and dry season). Dissolved organic carbon (DOC) and DON concentration and composition were measured by size-exclusion chromatography and by fluorescence measurements^16^,^17^,^18^. Based on the fluorescence measurements, five fluorophores were modelled (C1-C5) by parallel factor analysis^18^. Dissolved inorganic nitrogen (DIN, nitrate + nitrite and ammonium) concentration was also measured in all samples.

## Results

**Figure 1:**
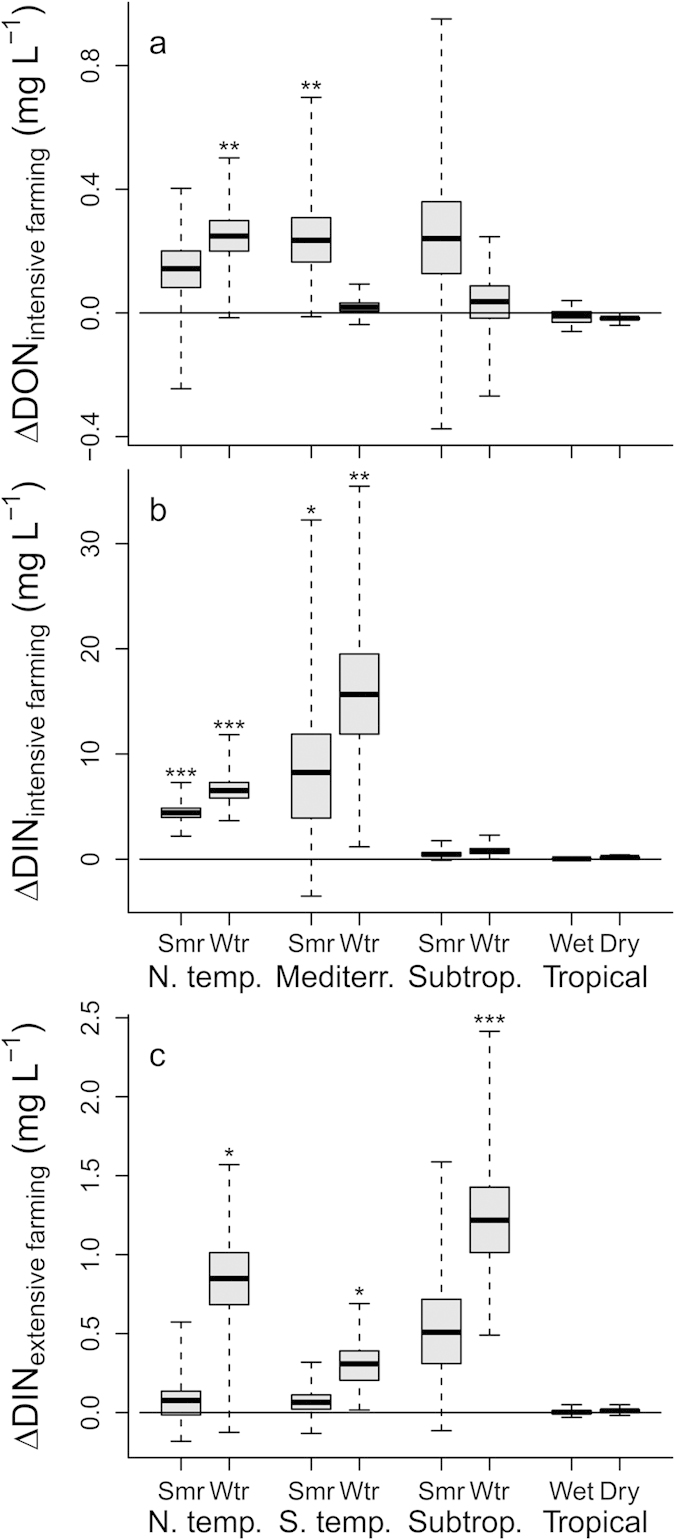
Effects of agriculture on fluvial dissolved nitrogen concentrations. Effect of intensive farming on dissolved organic nitrogen concentrations (panel (**a**)) and both intensive farming and extensive farming on dissolved inorganic nitrogen concentrations (panels (**b**,**c**) DIN = sum of nitrate and ammonium) concentrations. Due to lacking significance, the effects on DON are not shown for extensive farming. Boxplots show median (line), interquartile range (box) and data extremes (whiskers). The errors of the differences were calculated as bootstrap standard errors. p were calculated by Monte-Carlo resampling tests: ***p < 0.001 **p < 0.01, *p < 0.05; Smr = Summer, Wtr = Winter, N. temp. = northern temperate, S. temp. = southern temperate, Mediterr. = Mediterranean, Subtrop. = subtropical.

Across climate zones, intensive farming resulted in a general significant increase of DON and DIN concentrations relative to the reference catchments (*p* < 0.001, Monte-Carlo resampling test). Separate analyses of the individual climate zones show that the effect of intensive farming on DON and DIN concentrations was largely driven by the catchments in northern temperate and Mediterranean climate (Fig. 1a,b). Moreover, DIN concentration increased significantly in catchments with extensive farming, but to a lesser extend than for intensive farming (*p* < 0.001, Fig. 1c). No general effect of intensive farming on DOC concentrations or of extensive farming on DOC or DON concentrations was found (*p* > 0.05).

Both intensive farming (*p* < 0.001, permutational MANOVA) and extensive farming (*p* = 0.034) affected the molecular composition of DOM across climate zones. Separate tests of the individual climate zones show that intensive farming affected in-stream DOM composition in the northern temperate and Mediterranean climate (*p* < 0.001 and *p* = 0.002), and extensive farming affected in-stream DOM composition in the northern temperate and subtropical climate (*p* = 0.002 and *p* = 0.010). The described effects were found in both main seasons, indicating a stable source of altered fluvial DOM in the agricultural catchments.

**Figure 2:**
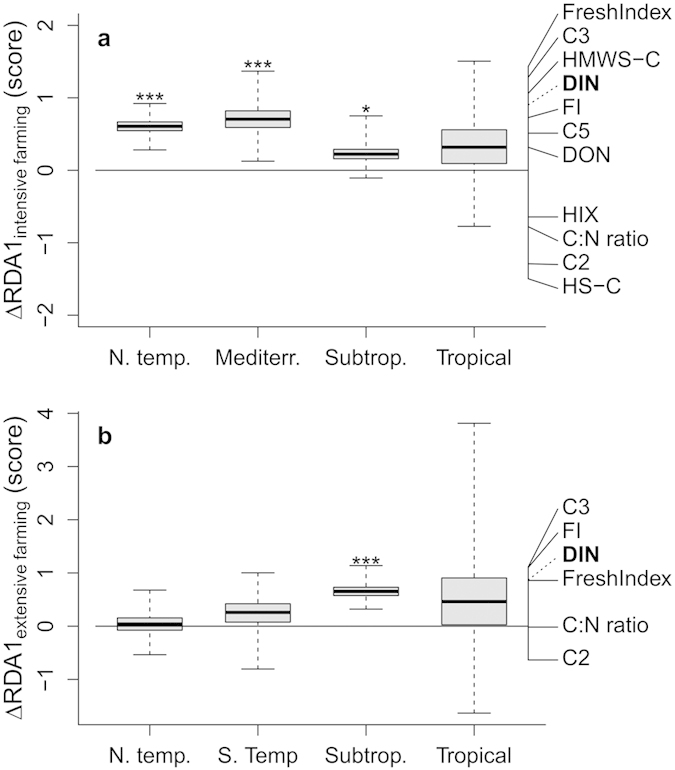
Effects of agriculture on fluvial dissolved organic matter composition. The shown scores and loadings are based on the first axis of a redundancy analysis (RDA1). Boxplots show median (line), interquartile range (box) and extremes (whiskers) of the effects for intensive farming (panel (**a**)) and extensive farming (panel (**b**)) on RDA1 scores for DOM. Stacked lines on the right show the loadings of DOM composition variables on RDA1 and the correlation to nitrate and ammonium (marked in bold, with dotted line). The errors of the differences were calculated as bootstrap standard errors. Asterisks indicate the climate zones, in which the scores of RDA1 where significantly affected by DIN: ***p < 0.001, *p < 0.05 (Spearman correlation). N. temp. = northern temperate, S. temp. = southern temperate, Mediterr. = Mediterranean, Subtrop. = subtropical, C2, C3, C5 = contribution of PARAFAC components 2, 3 & 5 to total sample fluorescence, HIX = humification index, FreshIndex = freshness index, HS-C = contribution of humic substances to DOC, HMWS-C = contribution of non-humic high-molecular weight substances to DOC, C:N ratio = C:N ratio of bulk DOM.

Across climate zones, fluvial DOM from agricultural catchments was generally more microbial in character and less characteristic of higher terrestrial plant sources than DOM from reference catchments. For intensive farming, this was evident from i) a higher fluorescence index (FI, *p* < 0.001, Monte-Carlo resampling test, Fig. 2a), indicating a more microbial source^19^; ii) a higher freshness index (FreshIndex, *p* < 0.001), indicating a rather recent, microbial DOM source^19^; iii) a lower humification index (HIX, *p* = 0.001), indicating less complex material^19^; iv) a lower C:N ratio (*p* < 0.001), indicating a lower content of refractory carbon from higher-plant sources^20^; v) more carbon in the proteinuous/polysacharide, high-molecular weight chromatographic fraction (HMWS-C, *p* < 0.001) and less carbon in the humic-like chromatographic fraction (HS-C, *p* = 0.019), indicating a shift from plant to microbial origin^21^; vi) more protein-like fluorescent DOM (fluorophore C5, *p* = 0.028) and a shift from plant-derived (fluorophore C2, *p* < 0.001) to microbially-derived fluorescent DOM (fluorophore C3, *p* < 0.001, Fig. 2b)^19^. For extensive farming, the effect was similar and evident from i) a higher FI (*p* = 0.028, Fig. 2b); ii) a higher FreshIndex (*p* = 0.013); iii) a lower C:N ratio (*p* = 0.013) and a shift from fluorophore C2 (*p* = 0.026) to fluorophore C3 (*p* = 0.035).

The aforementioned higher DON concentrations and the microbial-like character of fluvial DOM from intensive farming catchments was correlated to higher DIN concentrations (permutational MANOVA, *p* < 0.001, Fig. 2), whereas no overall relationship for the catchments with extensive farming was found (*p* = 0.42, Fig. 2). In detail, the changes of DOM composition and DON concentration in intensive farming were positively correlated to DIN concentrations in the subtropical, northern temperate and Mediterranean climates (*p* < 0.05, Spearman rank correlation, Fig. 2a). For extensive farming, these changes were only positively correlated to DIN concentrations in the subtropical climate zone (*p* < 0.05, Fig. 2b).

**Figure 3:**
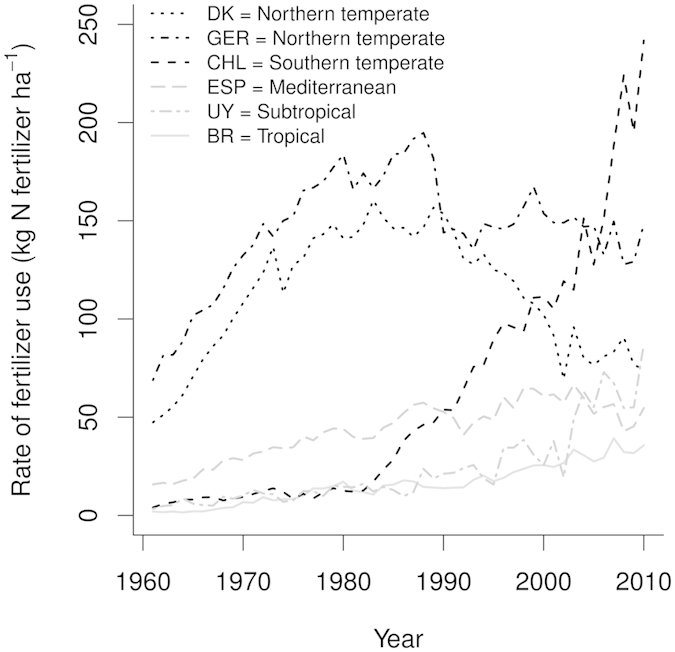
Intensity of total nitrogen fertilizer application in arable farming from 1961–2010. Data from country-wise data base of the FAO (http://faostat.fao.org). DK = Denmark, GER = Germany, CHL = Chile, ESP = Spain, URU = Uruguay, BR = Brazil.

The observed variation of the effect of agriculture on DIN and DOM (Figs 1 and 2) may be explained by the current and historical intensity of nitrogen fertilizer application in the investigated climate zones. This is supported by strong differences in the general temporal development of the intensity of intensive farming in the different countries (Fig. 3): The magnitude of the nitrogen fertiliser application in agriculture peaked in the 1980s for the countries sampled in the northern temperate climate (Germany and Denmark, Fig. 3). In all other countries a continuing increase is apparent, with Chile being the most extreme case (Fig. 3).

## Discussion

Intensive farming resulted in a concomitant increase of both DIN and DON concentrations in the investigated catchments. Furthermore, fluvial DOM from agricultural catchments was generally more microbial in character and less characteristic of higher terrestrial plant sources than DOM from reference catchments. Based on this, we conclude that the effect of agriculture on DIN concentrations is correlated to the effect of agriculture on DON concentrations and DOM composition. Here, higher DIN concentrations may not directly drive the changes in fluvial DOM. However, our results suggest that mechanisms related to intensification of agriculture could be the source of the relationship between increased DIN concentrations and changed character of fluvial DOM. Intensified soil tillage, drainage and fertilization, either as separate mechanisms or in combination, could affect the DOM exported to fluvial networks^7^,^8^.

The past and current fertilizer use intensity was high in the same regions in which we found the strongest effects of intensive farming on fluvial DIN concentrations, DON concentrations and DOM composition. This data supports the aforementioned idea that intensive agricultural management, including intensive fertilizer, the use of heavy machinery, intensive soil tillage, drainage and the intensive use of pesticides alters the soil microbial processing of DOM. Moreover, a further intensification of agriculture is expected in developing countries, since in addition to population growth, the per capita food demand will increase with increasing gross-domestic product in the future^22^. Therefore, the strong effects of intensive farming on fluvial DOM composition which we found in the northern temperate climate are a potential future scenario in regions with a currently lower intensification of agriculture.

Due to the spatial extent of agriculture^15^ and its contribution to anthropogenic carbon losses^4^,^5^, it can be assumed that fluvial DOM from agriculture is a major carbon source to aquatic ecosystems. Moreover, according to laboratory studies it is likely that agricultural DOM with higher contents of DON is of higher reactivity and will be mineralized faster than DOM from comparable reference catchments^6^,^14^. Hence, global intensification of agriculture may result in the release of large amounts of biogeochemically reactive DOM to fluvial networks, thereby altering the biogeochemical cycles related to DOM and increasing the productivity, respiration and outgassing of CO_2_ from fluvial networks on a global scale.

## Methods

### Sampling

We sampled headwater streams in catchments with a size ranging between 0.1–46.6 km^2^ (Supplementary Catchment Data). Catchments selected for dominant arable farming or livestock production, exhibited the respective land use on >50% of their area (Supplementary Catchment Data). In reference catchments, the reference vegetation type covered ≥60% of the area (Supplementary Catchment Data). Water samples were immediately filtered through 0.45 *μ*m filters and frozen within 24 h for transport. All samples were analysed at the Leibniz-Institute of Freshwater Ecology and Inland Fisheries in Berlin.

### Spectroscopic analyses of DOM

Excitation was measured from 240–450 nm in 5 nm steps and emission was measured from 300–600 nm in 2 nm steps. Both were measured with a bandwidth of 5 nm and a speed of 700–1500 nm s^−1^ depending on the sample concentration, using a Perkin-Elmer LS-50B fluorescence spectrometer (Rodgau, Germany). All samples were measured at room temperature. To correct for inner-filter effects, absorbance was measured on a Shimadzu UV-2401 UV/Vis spectrophotometer (Duisburg, Germany), using the same 1 cm quartz glass cuvettes as used for the fluorescence measurements.

We used the drEEM toolbox to standardise all measured excitation-emission-matrixes (EEMs)^18^: In detail, spectral correction was conducted based on instrument-specific values for excitation and by a correction kit for emission (BAM fluorescence calibration kit)^23^. Inner-filter effect correction was conducted based on absorbance measurements^18^. All samples were Raman-normalized based on measurements of the Raman peak at 350 nm.

Based on the fluorescence measurements, three indices were calculated: i) the fluorescence index (FI), which indicates a more microbial (FI ~ 1.9) or a more terrestrial higher plant (FI ~ 1.4) origin of the DOM^19^, ii) the freshness index (FreshIndex), which indicates the freshness of the material with values >1 representing freshly produced DOM, and values of 0.6–0.8 representing rather decomposed DOM and iii) the humification index for which higher values indicate more humified DOM^19^. A parallel factor analysis (PARAFAC) model with five components was validated by using residual and sum-of-squared-error investigation, as well as split-half validation (Supplementary Fig. S1) and random initialisation with 20 iterations^18^.

The character of the components was interpreted based on the fluorescence maxima and spectra (Supplementary Fig. S1 and Supplementary Table S1). C1 and C4 resembled terrestrial humic-like fluorophores exported ubiquitously from catchments^7^,^24^ and potentially susceptible to photodegradation^25^. C2 resembled a ubiquitous fulvic-like fluorophore and C3 a humic-like fluorophore dominating agricultural DOM^7^,^24^. The ratio of C2 and C3 was shown to indicate higher-plant (C2) or microbial (C3) sources of DOM^26^. A component similar to C3 was also linked to bacterial production and arable farming in a Canadian study^6^. C5 resembled a tryptophan-like fluorophore and is part of the protein-like fluorescence, which is positively related to the microbial availability of DOM^19^,^24^.

### Chromatographic analysis of DOM

Size-exclusion chromatography (SEC) was applied to analyse the molecular-size composition of DOC and DON, and the sum of the DOC and DON molecular-size fractions was used to represent the DOC and DON concentrations. The system used in this study was developed by Huber *et al.* (2011)^16^ and the direct measurement of DON with high accuracy was demonstrated in freshwaters for this SEC system by Graeber *et al.* (2012)^17^.

In SEC, a combination of UV- and IR- organic carbon detection and UV- organic nitrogen detection was used^16^,^17^. This procedure differentiated between non-humic high molecular weight substances (HMWS) of hydrophilic character (polysaccharides, proteins, amino sugars), humic-like substances (HS) with higher aromaticity based on UV measurements at 254 nm, and between low-molecular weight acids and neutrals which were combined as the low-molecular weight fraction in this study (Supplementary Fig. S2, LMWS)^16^,^17^. LMWS referred to neutral, hydrophilic to amphiphillic substances (alcoholes, aldehydes, ketones, sugars, amino acids)^16^. The humic-like substance fraction in SEC had a similar column retention time as humic and fulvic substance extracts provided by the International Humic Substance Society^16^. The DON measured by SEC did not include the LMWS fraction, since it could not accurately be differentiated from nitrate^16^. This fraction contains very little DON in natural freshwaters, usually not affecting DON determination with SEC^17^.

### Analysis of dissolved inorganic nitrogen

Nitrate (measured as nitrate plus nitrite) and ammonium concentrations were measured by standard spectrophotometric methods (ISO 13395 and ISO 11732).

### Statistics

Since assumptions for parametric statistics often were not fulfilled, non-parametric tests were conducted.

Monte–Carlo resampling tests of the effect of land use on DIN, DOC and DON concentrations and single variables of DOM composition were conducted with the coin package^27^ in R (version 3.0^28^) using the interaction of climate zone and season as stratum (block). Permutational MANOVAs of the effect of land use on DOM composition were conducted with the vegan package^29^ in R, based on Euclidean distances and climate zone and season as strata. When the effects of land use in single climate zones were analysed by Monte–Carlo resampling tests and permutational MANOVAs, season was used as stratum. All tests were conducted with 9999 iterations.

The redundancy analysis (RDA) on the relationship of DOM composition to the DIN concentration (sum of nitrate + nitrite and ammonium) was performed with the rda function (vegan package) as partial RDA, with climate zone and season as constraints. Significance of the RDA model, axes and terms was tested with permutational ANOVAs of the vegan package^29^. Permutational Spearman tests were conducted on the RDA site scores from RDA axis 1 with Monte–Carlo resampling (coin package). All tests were conducted with 9999 iterations.

## Additional Information

**How to cite this article**: Graeber, D. *et al.* Global effects of agriculture on fluvial dissolved organic matter. *Sci. Rep.* **5**, 16328; doi: 10.1038/srep16328 (2015).

## Supplementary Material

Supplementary Information
