# Supplementary material for: Global effects of agriculture on fluvial dissolved organic matter
Source: Sci Rep. 2015 Nov 6;5:16328. doi: 10.1038/srep16328 (PMC4635380; doi:10.1038/srep16328)
Supplement: Supplementary Information [file srep16328-s1.pdf]

Global effects of agriculture on fluvial dissolved organic matter

Daniel Graeber[1,*], Iola G. Boëchat[2], Francisco Encina-Montoya[3], Carlos Esse[3], Jörg Gelbrecht[4], Guillermo Goyenola[5], Björn Gücker[2], Marlen Heinz[6], Brian Kronvang[1], Mariana Meerhoff[1,5], Jorge Nimptsch[7], Martin T. Pusch[6], Ricky C. S. Silva[8], Daniel von Schiller[9], and Elke Zwirnmann[4]

[1]Department of Bioscience, Aarhus University, Denmark
[2]Department of Geosciences, Federal University of São João del-Rei, Brazil
[3]Faculty of Natural Resources, Environmental Science Nucleus, Catholic University of Temuco, Chile
[4]Department of Chemical Analytics and Biogeochemistry, Leibniz-Institute of Freshwater Ecology and Inland Fisheries, Germany
[5]Department of Theoretical and Applied Ecology, CURE – Faculty of Sciences, Universidad de la República, Uruguay
[6]Department of Ecosystem Research, Leibniz-Institute of Freshwater Ecology and Inland Fisheries, Germany
[7]Institute of Marine and Limnological Sciences, Faculty of Sciences, University Austral de Chile, Chile
[8]Department of Natural Sciences, Federal University of São João del-Rei, Brazil
[9]Department of Plant Biology and Ecology, Faculty of Science and Technology, University of the Basque Country, Spain
[*]dgr@bios.au.dk , Second to last author are ordered in alphabetical order.

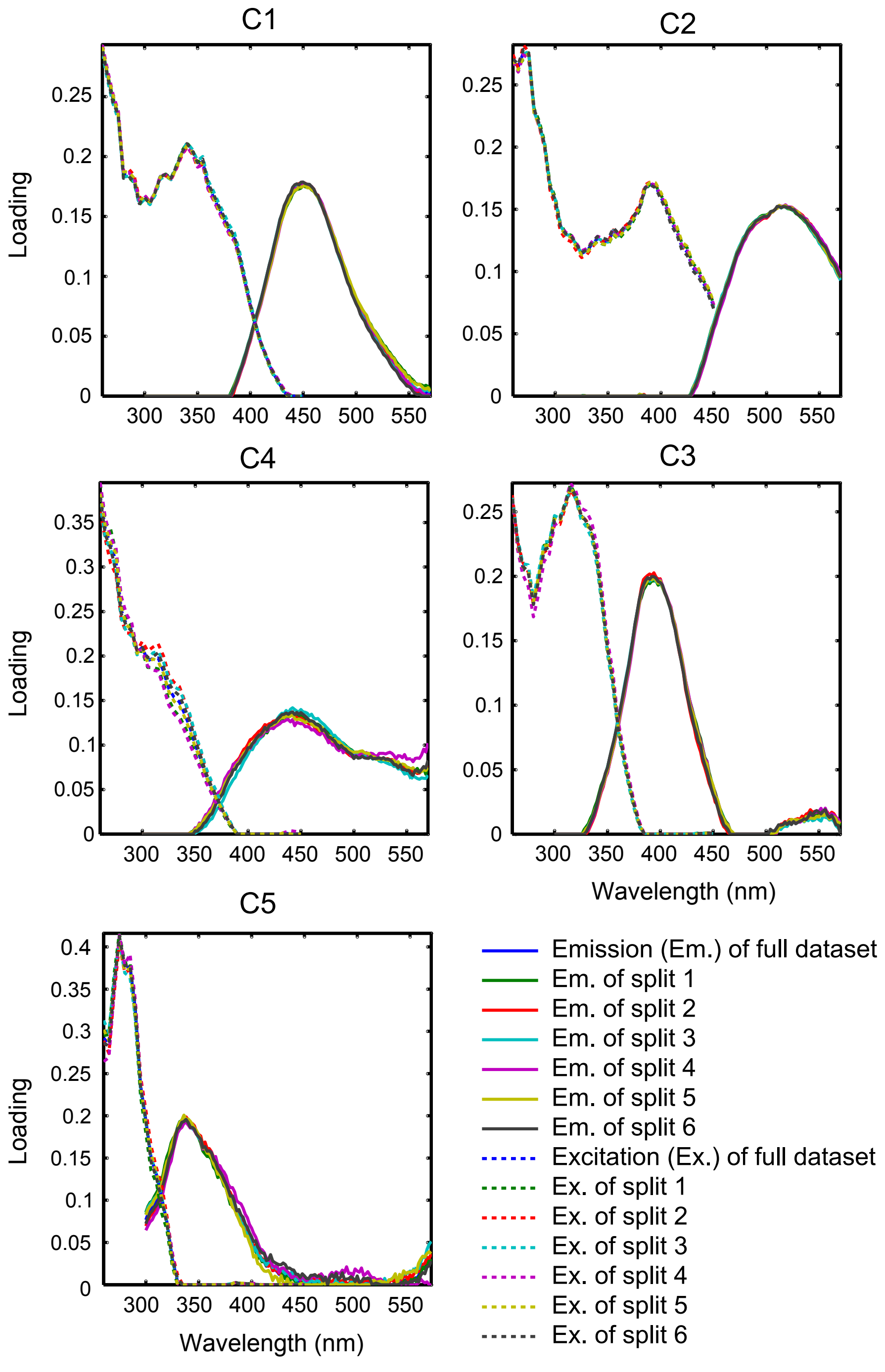

Supplementary Figure S1 | Split-half validation of the PARAFAC model. The models for six halfs generated by split-half validation are shown. When the fits of the splits are similar to each other and the entire model, a high stability of the model and low randomness of the fluorophores is given.

Supplementary Table S1 | Maxima of the PARAFAC components (nm).

Component	Primary Ex. peak	Secondary Ex. peak	Emission peak
C1	< 260	340	450
C2	270	390	518
C3	315	< 260	394
C4	< 260	–	442
C5	275	–	336

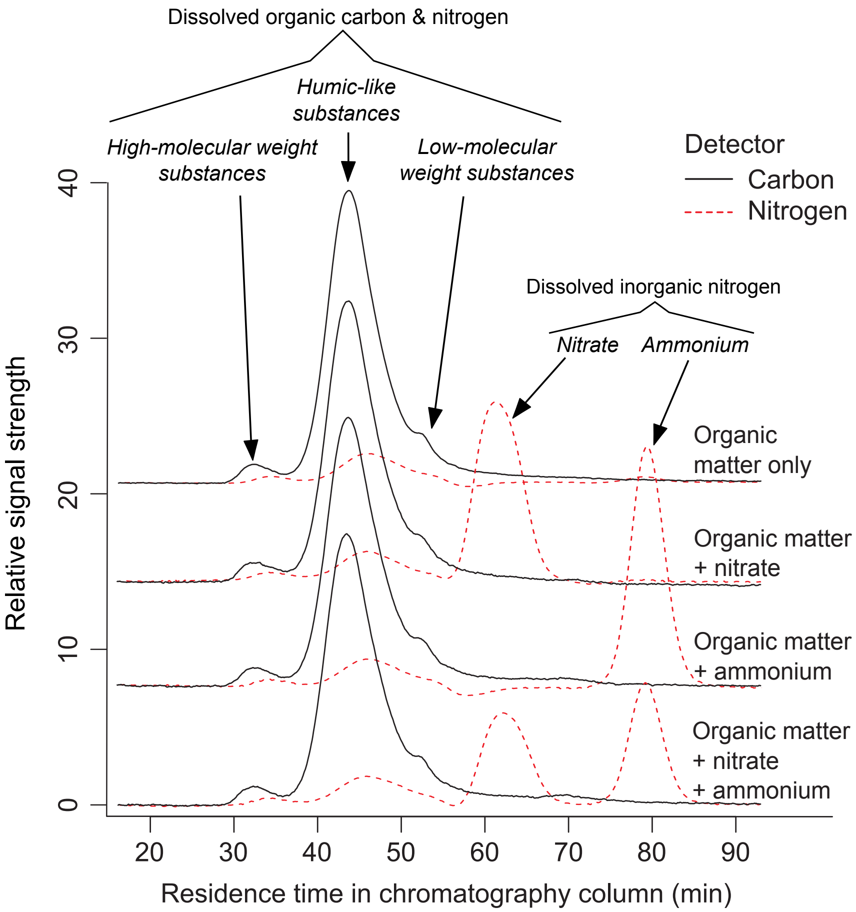

Supplementary Figure S2 | Typical chromatogram of size-exclusion chromatography. The chromatogram shows the distribution of DOM fractions with and without added nitrate and ammonium. The sample for this chromatogram was taken at a wetland outflow (part of the German subdataset of this study) .

Supplementary Catchment Data

The number of sampled catchments ranged between 4 and 19 for each pristine, intensive farming and extensive farming catchments within each season and climate zone (Supplementary Table S2). Excepted from this were tropical catchments, as due to very low DOM concentrations, the DOM composition measurements for some samples exhibited a high uncertainty and thus had to be excluded. Therefore, only two reference catchments could be included for each dry and wet season and only three arable farming catchments could be included for the dry season (Supplementary Table S2). In addition, one stream could only be sampled once due to seasonal stream intermittency (ESP-06). Other streams of the same land use were sampled instead. Moreover, the summer samples for DK-13 and GER-05 and a part of the samples from Chile (7 samples from winter/wet season: CHL-10, CHL-11, CHL-12, CHL-13, CHL-14, CHL-15, CHL-17) and Uruguay (2 samples from wet season: DF-03 and DF-08) were damaged during transport and thus could not be measured. These samples represented only one observation within one of the seasons (Supplementary information Table S2).

Detailed soil and land use characteristics are available for the countries of the northern temperate (Germany, Denmark), Mediterranean (Spain) and southern temperate (Chile) climate zone (Supplementary information Table S3, S4, S5). Here the percentages of agricultural land-use types could be analysed in detail based on the European Corine dataset[1] in Germany and Spain, based on a national mapping in Denmark, and based on Landsat data in Chile an Uruguay. Landsat data was also planned to be used for Brazil, however, the land use classification of the Landsat data were in disagreement with the data from ground-based surveys at the streams. Thus, we used classes based on ground surveys for the land use groupings instead of the Landsat data for Brazil.

References

[1] European Environmental Agency. Clc2006 technical guidelines. Tech. Rep., European Environmental Agency (2007).

Supplementary Table S2 | Site abbreviations, climate zone, country, number of observations, catchment size and GPS coordinates. Each site was sampled during the two main seasons, but due to different reasons, a part of the sites could only be sampled once (see text above for explanations). Pristine, pristine forest and pristine wetland belong to the reference catchments.

Site	Biome	Country	Type	Observations	Catchment size	GPS Y	GPS X
					km2	WGS 84, decimal degrees	
DK-01	Northern temperate	Denmark	intensive farming	2	14.4	55.65	8.53
DK-02	Northern temperate	Denmark	intensive farming	2	9.5	55.49	9.2
DK-03	Northern temperate	Denmark	intensive farming	2	6	56	9.85
DK-04	Northern temperate	Denmark	intensive farming	2	46.4	56.11	9.82
DK-05	Northern temperate	Denmark	intensive farming	2	3.9	56.23	9.76
DK-07	Northern temperate	Denmark	pristine	2	4.6	56.08	9.42
DK-09	Northern temperate	Denmark	pristine	2	0.6	56.25	10.6
DK-10	Northern temperate	Denmark	intensive farming	2	11.2	56.64	9.04
DK-11	Northern temperate	Denmark	pristine	2	5.4	56.47	8.38
DK-12	Northern temperate	Denmark	intensive farming	2	21.9	55.84	9.51
DK-13	Northern temperate	Denmark	pristine	1	0.4	55.11	10.32
DK-14	Northern temperate	Denmark	pristine	2	0.6	55.09	10.54
DK-15	Northern temperate	Denmark	intensive farming	2	4.3	55.12	10.77

Continued on next page

Site	Biome	Country	Type	Observations	Catchment size	GPS Y	GPS X
					km2	WGS 84, decimal degrees	
DK-16	Northern temperate	Denmark	intensive farming	2	5.4	55.8	12.03
DK-17	Northern temperate	Denmark	pristine	2	6.1	55.96	12.35
DK-18	Northern temperate	Denmark	pristine	2	0.6	55.78	12.44
DK-19	Northern temperate	Denmark	intensive farming	2	12.9	55.49	11.77
DK-20	Northern temperate	Denmark	intensive farming	2	15	55.33	11.6
DK-21	Northern temperate	Denmark	pristine	2	0.5	56.12	9.52
GER-02	Northern temperate	Germany	intensive farming	2	4.1	52.18	14.18
GER-03	Northern temperate	Germany	intensive farming	2	3.5	52.34	14.35
GER-04	Northern temperate	Germany	intensive farming	2	2.1	52.15	14.13
GER-05	Northern temperate	Germany	intensive farming	1	3.5	52.17	14.13
GER-06	Northern temperate	Germany	intensive farming	2	3.9	52.15	14.11
GER-07	Northern temperate	Germany	intensive farming	2	4.3	52.44	14.26
GER-08	Northern temperate	Germany	pristine forest	2	5	52.26	14.07
GER-09	Northern temperate	Germany	intensive farming	2	8.9	52.41	14.21
GER-10	Northern temperate	Germany	intensive farming	2	2.5	52.44	14.14
GER-11	Northern temperate	Germany	pristine forest	2	0.1	52.13	14.47

Continued on next page

Site	Biome	Country	Type	Observations	Catchment size	GPS Y	GPS X
					km2	WGS 84, decimal degrees	
GER-12	Northern temperate	Germany	pristine forest	2	1.3	52.1	14.49
GER-13	Northern temperate	Germany	pristine forest	2	0.3	52.11	14.43
GER-14	Northern temperate	Germany	extensive farming	2	20.2	52.76	12.94
GER-15	Northern temperate	Germany	extensive farming	2	8.7	52.78	13.04
GER-17	Northern temperate	Germany	extensive farming	2	3.5	52.29	13.33
GER-18	Northern temperate	Germany	extensive farming	2	4.4	52.29	13.33
GER-20	Northern temperate	Germany	extensive farming	2	24.3	52.78	12.94
GER-21	Northern temperate	Germany	pristine forest	2	1.7	52.11	14.43
GER-22	Northern temperate	Germany	pristine wetland	2	2.4	52.35	14.2
GER-23	Northern temperate	Germany	pristine wetland	2	6.6	52.52	13.84
GER-24	Northern temperate	Germany	pristine wetland	2	0.4	52.47	13.97
GER-25	Northern temperate	Germany	pristine wetland	2	1.2	52.47	13.97
GER-26	Northern temperate	Germany	pristine wetland	2	0.2	52.34	13.8
GER-28	Northern temperate	Germany	pristine wetland	2	6.8	52.16	13.58
ESP-01	Mediterranean	Spain	pristine	2	8.1	41.71	2.61
ESP-02	Mediterranean	Spain	pristine	2	13.2	41.7	2.58

Continued on next page

Site	Biome	Country	Type	Observations	Catchment size	GPS Y	GPS X
					km2	WGS 84, decimal degrees	
ESP-03	Mediterranean	Spain	pristine	2	1.4	41.71	2.56
ESP-04	Mediterranean	Spain	pristine	2	4.7	41.71	2.56
ESP-05	Mediterranean	Spain	pristine	2	0.7	41.66	2.53
ESP-06	Mediterranean	Spain	intensive farming	1	1.4	42.08	2.83
ESP-07	Mediterranean	Spain	intensive farming	2	1.1	42.09	2.84
ESP-08	Mediterranean	Spain	pristine	2	11.5	42.22	2.8
ESP-09	Mediterranean	Spain	intensive farming	2	0.8	42.24	2.86
ESP-10	Mediterranean	Spain	intensive farming	2	1.2	42.24	2.85
ESP-12	Mediterranean	Spain	intensive farming	2	1	42.22	3.06
ESP-13	Mediterranean	Spain	intensive farming	2	1	42.22	3.06
ESP-14	Mediterranean	Spain	pristine	2	19.1	41.83	2.94
BR-01	Tropical	Brazil	pristine	2	2.8	-21.06	-44.19
BR-02	Tropical	Brazil	pristine	1	1.4	-21.11	-44.18
BR-04	Tropical	Brazil	pristine	1	0.1	-21.04	-44.15
BR-11	Tropical	Brazil	intensive farming	1	0.2	-21.02	-44.23
BR-12	Tropical	Brazil	intensive farming	2	4.2	-21.17	-44.05

Continued on next page

Site	Biome	Country	Type	Observations	Catchment size	GPS Y	GPS X
					km2	WGS 84, decimal degrees	
BR-13	Tropical	Brazil	intensive farming	1	1.5	-21.04	-44.22
BR-14	Tropical	Brazil	intensive farming	2	4.2	-21.1	-44.2
BR-15	Tropical	Brazil	intensive farming	2	1.5	-20.99	-44.19
BR-16	Tropical	Brazil	extensive farming	2	2.1	-20.94	-44.06
BR-17	Tropical	Brazil	extensive farming	1	1.1	-21.06	-44.17
BR-18	Tropical	Brazil	extensive farming	1	1.4	-21	-44.19
BR-19	Tropical	Brazil	extensive farming	2	1.9	-21.02	-44.18
BR-20	Tropical	Brazil	extensive farming	2	0.2	-21.05	-44.08
BR-21	Tropical	Brazil	intensive farming	1	0.7	-21.07	-44.16
BR-22	Tropical	Brazil	extensive farming	1	0.5	-21.12	-44.2
CHL-02	Southern temperate	Chile	pristine	1	13.14	-38.55	-72.57
CHL-03	Southern temperate	Chile	extensive farming	2	28.51	-40.53	-72.59
CHL-04	Southern temperate	Chile	pristine	1	17.58	-40.28	-73.29
CHL-05	Southern temperate	Chile	pristine	2	13.61	-40.2	-73.42
CHL-06	Southern temperate	Chile	pristine	1	24.85	-40.23	-73.59
CHL-07	Southern temperate	Chile	pristine	2	9.05	-40.09	-73.37

Continued on next page

Site	Biome	Country	Type	Observations	Catchment size	GPS Y	GPS X
					km2	WGS 84, decimal degrees	
CHL-08	Southern temperate	Chile	pristine	2	24.89	-40.03	-73.03
CHL-09	Southern temperate	Chile	pristine	2	13.15	-39.33	-73.19
CHL-10	Southern temperate	Chile	extensive farming	1	34.37	-38.99	-73.21
CHL-11	Southern temperate	Chile	extensive farming	1	26.63	-38.96	-73.09
CHL-12	Southern temperate	Chile	extensive farming	1	34.27	-38.85	-73.04
CHL-13	Southern temperate	Chile	extensive farming	1	37.13	-39.15	-72.41
CHL-14	Southern temperate	Chile	extensive farming	1	38.78	-39.27	-72.47
CHL-15	Southern temperate	Chile	pristine	1	24.29	-39.41	-72.56
CHL-16	Southern temperate	Chile	extensive farming	2	27.26	-39.02	-72.46
CHL-17	Southern temperate	Chile	extensive farming	1	37.95	-40.34	-72.64
CHL-18	Southern temperate	Chile	extensive farming	2	26.62	-40.23	-72.61
A01	Subtropical	Uruguay	intensive farming	2	9.23	-34.16	-56.68
A12	Subtropical	Uruguay	intensive farming	2	12.31	-32.22	-54.43
A22	Subtropical	Uruguay	intensive farming	2	5.33	-34.06	-58.09
A23	Subtropical	Uruguay	intensive farming	2	4.32	-34.06	-58.06
A37	Subtropical	Uruguay	intensive farming	2	9.1	-33.48	-57.86

Continued on next page

Site	Biome	Country	Type	Observations	Catchment size	GPS Y	GPS X
					km2	WGS 84, decimal degrees	
A39	Subtropical	Uruguay	intensive farming	2	10.12	-33.16	-57.62
A58	Subtropical	Uruguay	intensive farming	2	9.39	-33.17	-58.12
A59	Subtropical	Uruguay	intensive farming	2	10.64	-33.17	-58.25
A60	Subtropical	Uruguay	intensive farming	2	7.69	-33.21	-58.31
A61	Subtropical	Uruguay	intensive farming	2	10	-33.15	-58.27
A63	Subtropical	Uruguay	intensive farming	2	21.7	-33.03	-57.95
A65	Subtropical	Uruguay	intensive farming	2	9.53	-33	-58.03
DF01	Subtropical	Uruguay	extensive farming	2	30.44	-34.06	-56.62
DF02	Subtropical	Uruguay	extensive farming	2	6.48	-34.13	-56.65
DF03	Subtropical	Uruguay	extensive farming	1	8.71	-34.15	-56.7
DF06	Subtropical	Uruguay	extensive farming	2	16.16	-33.22	-57.97
DF07	Subtropical	Uruguay	extensive farming	2	5.63	-32.81	-57.4
DF08	Subtropical	Uruguay	extensive farming	1	8.72	-32.86	-57.39
DF09	Subtropical	Uruguay	extensive farming	2	5.44	-32.8	-57.39
DF10	Subtropical	Uruguay	extensive farming	2	5.56	-32.79	-57.38
DF11	Subtropical	Uruguay	extensive farming	2	17.2	-34.18	-57.83

Continued on next page

Site	Biome	Country	Type	Observations	Catchment size	GPS Y	GPS X
					km2	WGS 84, decimal degrees	
E02	Subtropical	Uruguay	pristine	2	12.72	-33.98	-56.48
E05	Subtropical	Uruguay	pristine	2	20.93	-32.65	-56.54
E07	Subtropical	Uruguay	pristine	2	17.51	-32.33	-56.25
E08	Subtropical	Uruguay	pristine	2	3.06	-32.17	-56.11
E09	Subtropical	Uruguay	pristine	2	5.33	-32.21	-56.08
E30	Subtropical	Uruguay	pristine	2	6.31	-32.53	-56.63
E26	Subtropical	Uruguay	pristine	2	17.82	-32.57	-54.41
EFM	Subtropical	Uruguay	pristine	2	28.54	-31.79	-55.65

Supplementary Table S3 | Site abbreviations and land use in the catchments.

Site	Intensive farming	Extensive farming	Pristine	Other
		%		
DK-01	85.5	0.5	8.2	5.8
DK-02	92.7	0	0.5	6.8
DK-03	75.6	0.1	17.6	6.7
DK-04	86.9	0.3	4.4	8.5
DK-05	93.3	0	2.8	3.9
DK-07	26.1	0.2	70.5	3.2
DK-09	0	0	98.2	1.8
DK-10	88	0	0.4	11.6
DK-11	1.7	0	93.9	4.5
DK-12	88.4	0.3	3.7	7.5
DK-13	0	0	98.8	1.2
DK-14	6.7	0	90.1	3.2
DK-15	88.2	0	1.2	10.5
DK-16	88.1	0	3.5	8.4
DK-17	10.4	0	81	8.6
DK-18	5.5	0	89.8	4.7
DK-19	75.1	0.3	15.7	8.9
DK-20	74.5	0.1	11.6	13.9
DK-21	0.2	0	98	1.8
GER-02	88.5	0	0.5	11
GER-03	99.7	0	0	0.3
GER-04	99.7	0.3	0	0
GER-05	100	0	0	0
GER-06	100	0	0	0

Continued on next page

Site	Intensive farming	Extensive farming	Pristine	Other
		%		
GER-07	100	0	0	0
GER-08	29	0	71	0
GER-09	83.1	5.8	9.6	1.5
GER-10	97.8	0	0	2.2
GER-11	0	0	95.1	4.9
GER-12	27.7	0	72.3	0
GER-13	0	0	100	0
GER-14	15.2	71.9	8.3	4.5
GER-15	8.6	59.8	27.9	3.7
GER-17	17.7	76.9	2	3.4
GER-18	0.4	99.2	0.4	0
GER-20	0	78.5	21.5	0
GER-21	0	0	100	0
GER-22	0	0	100	0
GER-23	3.7	23.6	69	3.7
GER-24	0	0	86.8	13.2
GER-25	0	0	100	0
GER-26	0	0	100	0
GER-28	3.5	3.8	64.4	28.3
ESP-01	0.5	0	92.8	6.7
ESP-02	2.3	0	97.3	0.3
ESP-03	0	0	100	0
ESP-04	0.2	0.7	98.6	0.5
ESP-05	1.4	0	93.9	4.7
ESP-06	77.7	0	15.5	6.8

Continued on next page

Site	Intensive farming	Extensive farming	Pristine	Other
		%		
ESP-07	86.9	0	13.1	0
ESP-08	13.4	0	86.6	0
ESP-09	96.7	0	3.3	0
ESP-10	88.1	0	0	11.9
ESP-12	95	0	0	5
ESP-13	95	0	0	5
ESP-14	4.9	0	91.6	3.5
BR-01	0	0	> 80%	0
BR-02	0	0	> 80%	0
BR-04	0	0	> 80%	0
BR-11	> 50%	0	0	0
BR-12	> 50%	0	0	0
BR-13	> 50%	0	0	0
BR-14	> 50%	0	0	0
BR-15	> 50%	0	0	0
BR-16	0	> 50%	0	0
BR-17	0	> 50%	0	0
BR-18	0	> 50%	0	0
BR-19	0	> 50%	0	0
BR-20	0	> 50%	0	0
BR-21	> 50%	0	0	0
BR-22	0	> 50%	0	0
CHL-02	2	31.6	66.1	0.4
CHL-03	3	86	10.6	0.4
CHL-04	0	2.9	97.1	0

Continued on next page

Site	Intensive farming	Extensive farming	Pristine	Other
		%		
CHL-05	0	0	100	0
CHL-06	0	0	100	0
CHL-07	0	30.7	69.3	0
CHL-08	1.2	5.3	93.5	0
CHL-09	0	13.4	86.6	0
CHL-10	3.4	73.2	23.4	0
CHL-11	4.1	50.1	45.3	0.5
CHL-12	0.5	69.3	30.2	0
CHL-13	0.2	94.4	5.4	0
CHL-14	0	88	12	0
CHL-15	5.6	2.1	92.3	0
CHL-16	5.7	78.9	15	0.4
CHL-17	1.9	90	7.5	0.6
CHL-18	1.7	73.4	24.7	0.2
A01	100	0	0	0
A12	99.4	0	0	0.6
A22	91.1	0	8.3	0.6
A23	100	0	0	0
A37	74.7	0	24.9	0.3
A39	89.7	0	10.3	0.1
A58	>90	0	¡10	0
A59	93.6	0	1.3	5
A60	89.9	0	8.6	1.5
A61	88.8	0	8.7	2.6
A63	>90	0	¡10	0

Continued on next page

Site	Intensive farming	Extensive farming	Pristine	Other
		%		
A65	>90	0	¡10	0
DF01	0	98.6	0	1.4
DF02	0	100	0	0
DF03	0	99.7	0	0.3
DF06	0	99.4	0	0.6
DF07	0	98.8	0	1.2
DF08	0	99.7	0	0.3
DF09	0	98.8	0	1.2
DF10	0	99.9	0	0.1
DF11	0	97.8	1.2	1
E02	22.1	0	72.9	5
E05	0	0	99.4	0.6
E07	24.6	0	74.7	0.7
E08	0	0	100	0
E09	10.7	0	88.7	0.6
E30	8	0	91.9	0.2
E26	21.3	0	97.9	-19.1
EFM	3.7	0	90.6	5.7

Supplementary Table S4 | Site abbreviations and first part of soil types (Albeluvisols – Histosols) in the catchments. Please see Table S5 for first part.

Site	Albeluvisols	Andosols	Arenosols	Cambisols	Ferralsols	Fluvisols	Gleysols	Histosols
DK-01	0	0	0	0	0	0	0	0
DK-02	63.2	0	0	0	0	0	0	0
DK-03	0	0	0	0	0	0	0	0
DK-04	0	0	0	0	0	0	0	0
DK-05	0	0	0	0	0	0	0	0
DK-07	0	0	0	0	0	0	0	0
DK-09	0	0	100	0	0	0	0	0
DK-10	0	0	0	0	0	0	0	0
DK-11	0	0	0	0	0	0	0	0
DK-12	0	0	0	0	0	0	0	0
DK-13	0	0	0	0	0	0	0	0
DK-14	0	0	0	0	0	0	0	0
DK-15	0	0	0	0	0	0	0	0
DK-16	0	0	0	0	0	0	0	0
DK-17	0	0	0	0	0	0	0	0

Continued on next page

Site	Albeluvisols	Andosols	Arenosols	Cambisols	Ferralsols	Fluvisols	Gleysols	Histosols
DK-18	0	0	0	0	0	0	0	0
DK-19	0	0	12.8	87.2	0	0	0	0
DK-20	0	0	0	100	0	0	0	0
DK-21	0	0	0	0	0	0	0	0
GER-02	56.9	0	43.1	0	0	0	0	0
GER-03	96.3	0	3.7	0	0	0	0	0
GER-04	75.7	0	12	0	0	0	11.6	0.7
GER-05	47.6	0	52.4	0	0	0	0	0
GER-06	58.5	0	41.5	0	0	0	0	0
GER-07	94.8	0	0	0	0	0	0	5.2
GER-08	11.4	0	88.6	0	0	0	0	0
GER-09	73.7	0	13.1	0	0	0	6.9	0
GER-10	66.6	0	33.4	0	0	0	0	0
GER-11	0	0	100	0	0	0	0	0
GER-12	0	0	80.2	0	0	0	19.8	0
GER-13	0	0	100	0	0	0	0	0
GER-14	11.4	0	12.2	0	0	0	17.9	58.6

Continued on next page

Site	Albeluvisols	Andosols	Arenosols	Cambisols	Ferralsols	Fluvisols	Gleysols	Histosols
GER-15	0	0	27	0	0	0	9.2	63.7
GER-17	13	0	8.1	0	0	0	26.6	51.9
GER-18	0	0	0.8	0	0	0	0	99.2
GER-20	0	0	0	0	0	0	11.7	88.3
GER-21	0	0	99.8	0	0	0	0.2	0
GER-22	0	0	58.1	0	0	0	30.1	11.8
GER-23	0	0	74.1	0	0	0	0	25.9
GER-24	0	0	81.4	0	0	0	18.6	0
GER-25	0	0	63.2	0	0	0	36.8	0
GER-26	0	0	71.3	0	0	0	0	28.7
GER-28	0	0	67.8	0	0	0	14.3	17.2
ESP-01	0	0	0	0	0	0	0	0
ESP-02	0	0	0	0	0	1.4	0	0
ESP-03	0	0	0	0	0	100	0	0
ESP-04	0	0	0	0	0	66.4	0	0
ESP-05	0	0	0	0	0	0	0	0
ESP-06	0	0	0	49.2	0	0	0	0

Continued on next page

Site	Albeluvisols	Andosols	Arenosols	Cambisols	Ferralsols	Fluvisols	Gleysols	Histosols
ESP-07	0	0	0	100	0	0	0	0
ESP-08	0	0	0	73.2	0	0	0	0
ESP-09	0	0	0	100	0	0	0	0
ESP-10	0	0	0	100	0	0	0	0
ESP-12	0	0	0	0	0	100	0	0
ESP-13	0	0	0	0	0	100	0	0
ESP-14	0	0	0	0	0	73.6	0	0
BR-01	0	0	0	0	100	0	0	0
BR-02	0	0	0	0	100	0	0	0
BR-04	0	0	0	0	100	0	0	0
BR-11	0	0	0	0	100	0	0	0
BR-12	0	0	0	100	0	0	0	0
BR-13	0	0	0	0	100	0	0	0
BR-14	0	0	0	0	100	0	0	0
BR-15	0	0	0	0	100	0	0	0
BR-16	0	0	0	0	100	0	0	0
BR-17	0	0	0	0	100	0	0	0

Continued on next page

Site	Albeluvisols	Andosols	Arenosols	Cambisols	Ferralsols	Fluvisols	Gleysols	Histosols
BR-18	0	0	0	0	100	0	0	0
BR-19	0	0	0	0	100	0	0	0
BR-20	0	0	0	0	100	0	0	0
BR-21	0	0	0	0	100	0	0	0
BR-22	0	0	0	0	100	0	0	0
CHL-02	0	0	0	0	0	0	0	0
CHL-03	0	72.2	0	0	0	0	27.8	0
CHL-04	0	0	0	80.1	0	0	0	0
CHL-05	0	0	0	100	0	0	0	0
CHL-06	0	0	0	100	0	0	0	0
CHL-07	0	0	0	100	0	0	0	0
CHL-08	0	100	0	0	0	0	0	0
CHL-09	0	0	0	100	0	0	0	0
CHL-10	0	0	0	0	0	52.2	0	0
CHL-11	0	2.2	0	0	0	43	0	0
CHL-12	0	74.8	0	0	0	0.5	0	0
CHL-13	0	100	0	0	0	0	0	0

Continued on next page

Site	Albeluvisols	Andosols	Arenosols	Cambisols	Ferralsols	Fluvisols	Gleysols	Histosols
CHL-14	0	100	0	0	0	0	0	0
CHL-15	0	0	0	100	0	0	0	0
CHL-16	0	100	0	0	0	0	0	0
CHL-17	0	0	0	0	0	0	100	0
CHL-18	0	30.7	0	0	0	0	69.3	0
A01	0	0	0	0	0	0	0	0
A12	0	0	0	0	0	0	0	0
A22	0	0	0	0	0	0	0	0
A23	0	0	0	0	0	0	0	0
A37	0	0	0	0	0	0	0	0
A39	0	0	0	0	0	0	0	0
A58	0	0	0	0	0	0	0	0
A59	0	0	0	0	0	0	0	0
A60	0	0	0	0	0	0	0	0
A61	0	0	0	0	0	0	0	0
A63	0	0	0	0	0	0	0	0
A65	0	0	0	0	0	0	0	0

Continued on next page

Site	Albeluvisols	Andosols	Arenosols	Cambisols	Ferralsols	Fluvisols	Gleysols	Histosols
DF01	0	0	0	0	0	0	0	0
DF02	0	0	0	0	0	0	0	0
DF03	0	0	0	0	0	0	0	0
DF06	0	0	0	0	0	0	0	0
DF07	0	0	0	0	0	0	0	0
DF08	0	0	0	0	0	0	0	0
DF09	0	0	0	0	0	0	0	0
DF10	0	0	0	0	0	0	0	0
DF11	0	0	0	0	0	0	0	0
E02	0	0	0	0	0	0	0	0
E05	0	0	0	0	0	0	0	0
E07	0	0	0	0	0	0	0	0
E08	0	0	0	0	0	0	0	0
E09	0	0	0	0	0	0	0	0
E30	0	0	0	0	0	0	0	0
E26	0	0	0	0	0	0	0	0
EFM	0	0	0	0	0	0	0	0

Supplementary Table S5 | Site abbreviations and second part of soil types (Leptosols – Vertisols) in the catchments. Please see Table S4 for first part.

Site	Leptosols	Luvisols	Nitisols	Phaeozems	Planosols	Podzols	Regosols	Vertisols
DK-01	0	0	0	0	0	100	0	0
DK-02	0	0	0	0	0	36.8	0	0
DK-03	0	100	0	0	0	0	0	0
DK-04	0	61.2	0	0	0	38.8	0	0
DK-05	0	100	0	0	0	0	0	0
DK-07	0	0	0	0	0	100	0	0
DK-09	0	0	0	0	0	0	0	0
DK-10	0	100	0	0	0	0	0	0
DK-11	0	0	0	0	0	100	0	0
DK-12	0	92.1	0	0	0	7.9	0	0
DK-13	0	100	0	0	0	0	0	0
DK-14	0	100	0	0	0	0	0	0
DK-15	0	100	0	0	0	0	0	0
DK-16	0	7.3	0	0	0	92.7	0	0
DK-17	0	0	0	0	0	100	0	0

Continued on next page

Site	Leptosols	Luvisols	Nitisols	Phaeozems	Planosols	Podzols	Regosols	Vertisols
DK-18	0	100	0	0	0	0	0	0
DK-19	0	0	0	0	0	0	0	0
DK-20	0	0	0	0	0	0	0	0
DK-21	0	0	0	0	0	100	0	0
GER-02	0	0	0	0	0	0	0	0
GER-03	0	0	0	0	0	0	0	0
GER-04	0	0	0	0	0	0	0	0
GER-05	0	0	0	0	0	0	0	0
GER-06	0	0	0	0	0	0	0	0
GER-07	0	0	0	0	0	0	0	0
GER-08	0	0	0	0	0	0	0	0
GER-09	0	6.3	0	0	0	0	0	0
GER-10	0	0	0	0	0	0	0	0
GER-11	0	0	0	0	0	0	0	0
GER-12	0	0	0	0	0	0	0	0
GER-13	0	0	0	0	0	0	0	0
GER-14	0	0	0	0	0	0	0	0

Continued on next page

Site	Leptosols	Luvisols	Nitisols	Phaeozems	Planosols	Podzols	Regosols	Vertisols
GER-15	0	0	0	0	0	0	0	0
GER-17	0	0	0	0	0	0	0.3	0
GER-18	0	0	0	0	0	0	0	0
GER-20	0	0	0	0	0	0	0	0
GER-21	0	0	0	0	0	0	0	0
GER-22	0	0	0	0	0	0	0	0
GER-23	0	0	0	0	0	0	0	0
GER-24	0	0	0	0	0	0	0	0
GER-25	0	0	0	0	0	0	0	0
GER-26	0	0	0	0	0	0	0	0
GER-28	0	0	0	0	0	0	0.7	0
ESP-01	0	0	0	0	0	0	100	0
ESP-02	0	0	0	0	0	0	98.6	0
ESP-03	0	0	0	0	0	0	0	0
ESP-04	0	0	0	0	0	0	33.6	0
ESP-05	0	0	0	0	0	0	100	0
ESP-06	50.8	0	0	0	0	0	0	0

Continued on next page

Site	Leptosols	Luvisols	Nitisols	Phaeozems	Planosols	Podzols	Regosols	Vertisols
ESP-07	0	0	0	0	0	0	0	0
ESP-08	26.8	0	0	0	0	0	0	0
ESP-09	0	0	0	0	0	0	0	0
ESP-10	0	0	0	0	0	0	0	0
ESP-12	0	0	0	0	0	0	0	0
ESP-13	0	0	0	0	0	0	0	0
ESP-14	0	4.8	0	0	0	0	21.6	0
BR-01	0	0	0	0	0	0	0	0
BR-02	0	0	0	0	0	0	0	0
BR-04	0	0	0	0	0	0	0	0
BR-11	0	0	0	0	0	0	0	0
BR-12	0	0	0	0	0	0	0	0
BR-13	0	0	0	0	0	0	0	0
BR-14	0	0	0	0	0	0	0	0
BR-15	0	0	0	0	0	0	0	0
BR-16	0	0	0	0	0	0	0	0
BR-17	0	0	0	0	0	0	0	0

Continued on next page

Site	Leptosols	Luvisols	Nitisols	Phaeozems	Planosols	Podzols	Regosols	Vertisols
BR-18	0	0	0	0	0	0	0	0
BR-19	0	0	0	0	0	0	0	0
BR-20	0	0	0	0	0	0	0	0
BR-21	0	0	0	0	0	0	0	0
BR-22	0	0	0	0	0	0	0	0
CHL-02	0	0	100	0	0	0	0	0
CHL-03	0	0	0	0	0	0	0	0
CHL-04	0	0	19.9	0	0	0	0	0
CHL-05	100	0	0	0	0	0	0	0
CHL-06	100	0	0	0	0	0	0	0
CHL-07	27.6	0	0	0	0	72.4	0	0
CHL-08	0	0	0	0	0	0	0	0
CHL-09	0	0	0	0	0	0	0	0
CHL-10	0	0	47.8	0	0	0	0	0
CHL-11	0	0	54.8	0	0	0	0	0
CHL-12	0	0	24.7	0	0	0	0	0
CHL-13	0	0	0	0	0	0	0	0

Continued on next page

Site	Leptosols	Luvisols	Nitisols	Phaeozems	Planosols	Podzols	Regosols	Vertisols
CHL-14	0	0	0	0	0	0	0	0
CHL-15	0	0	0	0	0	0	0	0
CHL-16	0	0	0	0	0	0	0	0
CHL-17	0	0	0	0	0	0	0	0
CHL-18	0	0	0	0	0	0	0	0
A01	0	0	0	100	0	0	0	0
A12	0	0	0	100	0	0	0	0
A22	0	0	0	100	0	0	0	0
A23	0	0	0	100	0	0	0	0
A37	0	0	0	100	0	0	0	0
A39	0	0	0	100	0	0	0	0
A58	0	0	0	100	0	0	0	0
A59	0	0	0	100	0	0	0	0
A60	0	0	0	100	0	0	0	0
A61	0	0	0	100	0	0	0	0
A63	0	0	0	100	0	0	0	0
A65	0	0	0	100	0	0	0	0

Continued on next page

Site	Leptosols	Luvisols	Nitisols	Phaeozems	Planosols	Podzols	Regosols	Vertisols
DF01	0	0	0	100	0	0	0	0
DF02	0	0	0	100	0	0	0	0
DF03	0	0	0	100	0	0	0	0
DF06	0	0	0	100	0	0	0	0
DF07	0	0	0	100	0	0	0	0
DF08	0	0	0	100	0	0	0	0
DF09	0	0	0	100	0	0	0	0
DF10	0	0	0	100	0	0	0	0
DF11	0	0	0	100	0	0	0	0
E02	0	0	0	100	0	0	0	0
E05	0	0	0	0	0	0	0	100
E07	0	0	0	0	0	0	0	100
E08	0	0	0	0	0	0	0	100
E09	0	0	0	0	0	0	0	100
E30	0	0	0	0	0	0	0	100
E26	0	0	0	99	1	0	0	0
EFM	0	0	0	100	0	0	0	0
